# Imaging and AI based chromatin biomarkers for diagnosis and therapy evaluation from liquid biopsies

**DOI:** 10.1038/s41698-023-00484-8

**Published:** 2023-12-14

**Authors:** Kiran Challa, Daniel Paysan, Dominic Leiser, Nadia Sauder, Damien C. Weber, G. V. Shivashankar

**Affiliations:** 1 Mechano-Genomic Group, Division of Biology and Chemistry, Paul-Scherrer Institute, Villigen, Switzerland; 2 Department of Health Sciences and Technology, ETH Zurich, Zurich, Switzerland; 3 Center for Proton Therapy, Paul-Scherrer Institute, Villigen, Switzerland; 4 Department of Radio-Oncology, University Hospital Zurich, Zurich, Switzerland; 5 Department of Radio-Oncology, University of Bern, Bern, Switzerland

**Keywords:** Diagnostic markers, Prognostic markers, Cancer

## Abstract

Multiple genomic and proteomic studies have suggested that peripheral blood mononuclear cells (PBMCs) respond to tumor secretomes and thus could provide possible avenues for tumor prognosis and treatment evaluation. We hypothesized that the chromatin organization of PBMCs obtained from liquid biopsies, which integrates secretome signals with gene expression programs, provides efficient biomarkers to characterize tumor signals and the efficacy of proton therapy in tumor patients. Here, we show that chromatin imaging of PBMCs combined with machine learning methods provides such robust and predictive chromatin biomarkers. We show that such chromatin biomarkers enable the classification of 10 healthy and 10 pan-tumor patients. Furthermore, we extended our pipeline to assess the tumor types and states of 30 tumor patients undergoing (proton) radiation therapy. We show that our pipeline can thereby accurately distinguish between three tumor groups with up to 77% accuracy and enables the monitoring of the treatment effects. Collectively, we show the potential of chromatin biomarkers for cancer diagnostics and therapy evaluation.

## Introduction

Tumors arise in the stromal microenvironment composed of endothelial cells, blood vessels, immune cells alongside the extracellular matrix and soluble factors^1^. Importantly, the tumor cells secrete signals within the tumor microenvironment (TME) to stimulate their growth and progression^2,3^. Since the TME also consists of blood vessels, a variety of tumor factors and secreted signals also eventually enters the bloodstream^4^. The composition and concentration of the secretome signals depends strongly on the stage of the disease, suggesting that the therapeutic options such as e.g. chemotherapy, surgery or radiation therapy^5–7^ may alter the signal profiles.

Recent approaches have exploited the resulting presence of tumor signals in the bloodstream to develop a variety of cancer biomarkers primarily used for cancer diagnosis and evaluation of treatment efficacy^8,9^. For instance, cytokines, growth factors, exosomes, tumor specific DNA and circulating tumor cells found in the blood have been used as biomarkers^10–12^. Transcriptomic and proteomic analyses of blood cells have also provided additional biomarkers for cancer detection^13–18^ and disease diagnostics^19–24^. Despite their success, these methods often rely on resource-intensive sequencing and proteomic methods, are limited to late-stage diagnosis and process-intensive. Thus, novel patient-specific biomarkers for timely evaluation of tumor type and stage are required.

Recent studies highlight the important role of epigenetic chromatin remodeling in gene expression regulation^25,26^. Specific chromosome conformations in PBMCs are diagnostic tools for cancers^27–31^. PBMC activation involves extensive chromatin remodeling and DNA methylation changes^32–34^. The secretome signals sensed by CD8 + T cells exhibit dysfunctional states in solid tumors, and the observed chromatin state dynamics are reflected through surface protein-associated markers, leading to chromatin accessibility differences in specific gene loci^35^. An integrated framework combining single-cell RNA-sequencing, epigenomic SNP maps, and genome-wide association study data on immune cell types unveils how genetic variations influence disease development and the activation of specific immune populations^36^. In contrast to transcriptomic and proteomic biomarkers, the cellular chromatin organization can be captured via e.g. simple immunofluorescence imaging of PBMCs. Machine learning approaches combined with such fluorescent imaging^37–40^ have recently provided novel possibilities to detect even subtle, signal-induced chromatin structure alterations of PBMCs^37,41^.

In this study, we developed an imaging and AI-based pipeline to characterize the 3D chromatin organization in PBMCs obtained using liquid biopsies from patients undergoing proton therapy, before, during and after this treatment modality. Our analyses show that our pipeline provides a robust read-out of the chromatin organization of PBMCs to discriminate between healthy and cancer patients and also between different tumor groups. We further show that our pipeline can also be applied to assess the treatment efficacy of proton therapy administered for advanced tumors, thus showing the potential of our developed chromatin biomarkers for two closely linked clinical applications, namely diagnosis and treatment evaluation.

## Results

### An imaging and AI-based pipeline to quantify chromatin features for cancer diagnostics using liquid biopsies

**Fig. 1:**
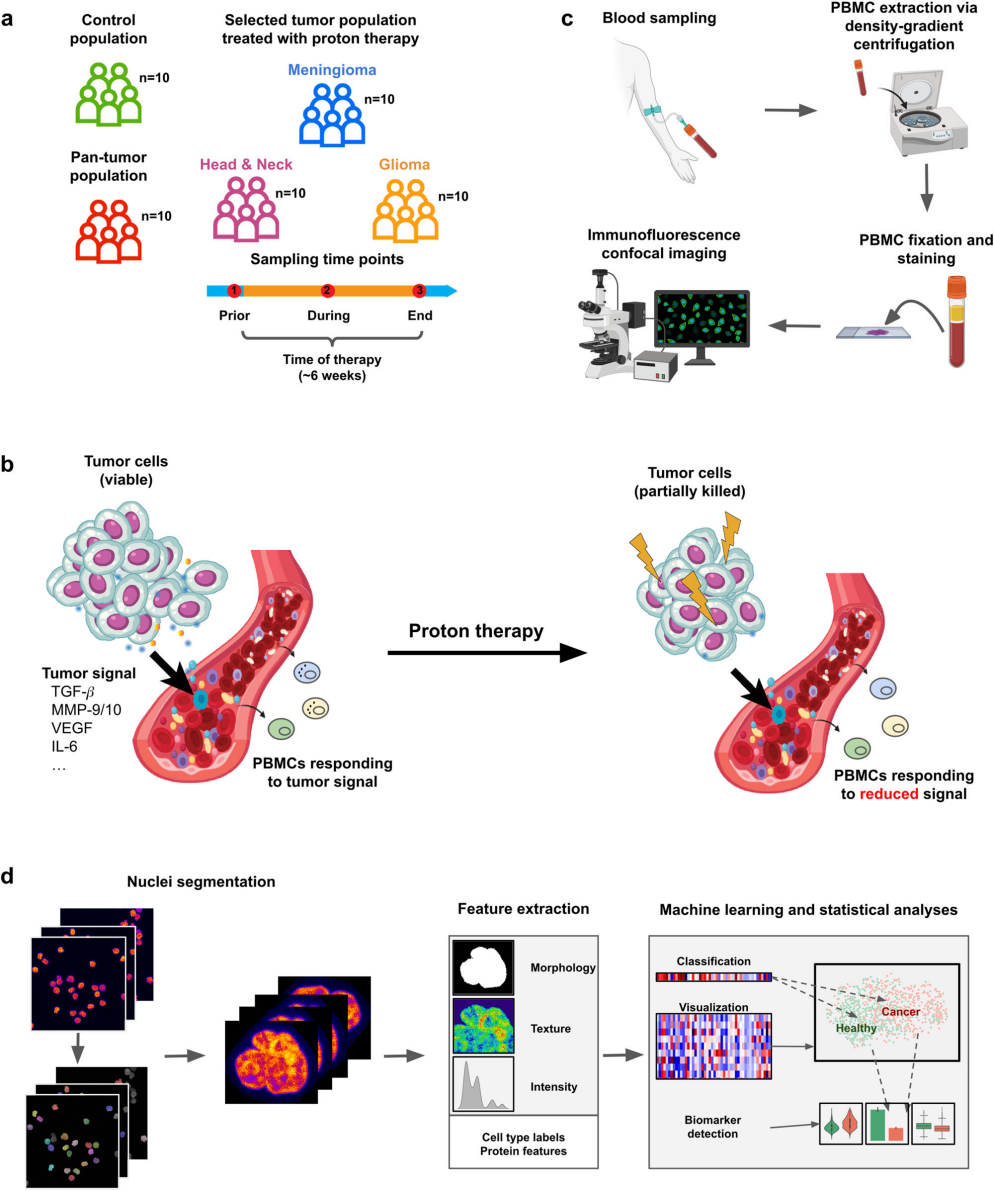
Overview of our platform to study the chromatin organization of peripheral blood mononuclear cells in a tumor and proton therapy setting. **a** Overview of the different study populations whose blood samples are analyzed in the context of the present work. In contrast to the pan-tumor and control population blood samples of meningioma, glioma and head and neck tumor patients undergoing proton therapy are obtained at three different time points: prior, during and at the end of the on average 6 week long therapy process. **b** Schematic representation of the effect of tumor signals on peripheral blood mononuclear cells (PBMCs). Secretome signals secreted by the tumor enter the bloodstream via blood vessels in the tumor microenvironment. The signals are sensed by PBMCs whose response involves chromatin reorganization. Successful therapy reduces the amount of viable tumor cells and the tumor signal concentration which eventually is also reflected in the chromatin organization of PBMCs exposed to the signals. Adapted with permission from ref. ^70^. **c** Schematic representation of the experimental sample processing. PBMCs are extracted from the obtained blood samples via density-gradient centrifugation, fixed and immunofluorescently stained and imaged using a confocal microscope. Created with BioRender.com. **d** Overview of our computational platform which first segments individual PBMCs from the input images to characterize the nuclear morphology and chromatin organization and measure the expression of selected protein markers as well as to derive related cell type labels. This information is used to study the chromatin organization of PBMCs in the selected tumor and therapy settings using statistical and machine learning-based analyses.

The pipeline uses cost-efficient single-cell fluorescent microscopy images obtained from non-invasive liquid biopsies from 10 healthy controls and 40 patients of various tumor groups that underwent proton therapy. Our pipeline identifies chromatin biomarkers that (a) enable accurate diagnostics of cancer and respective tumor subtypes and (b) allow for an immediate read-out of proton radiation-induced effects. In particular, we collected blood samples of patients undergoing proton therapy at three different time points during the treatment procedure (Fig. 1a). The therapy is expected to reduce the concentration and composition of the tumor secretome signals in the blood. These changing signals are sensed by the PBMCs of the patients and are thus reflected in their chromatin organization (Fig. 1b). To study these changes, the PBMCs are labeled with DAPI to stain chromatin, ɣH2AX (a DNA damage marker), Lamin A/C (a nuclear structural protein) and cell surface markers (CD3, CD4 and CD8) (Fig. 1c, see Methods). The resulting imaging information is used by our pipeline to identify chromatin alterations of PBMCs that differentiate healthy and tumor patients, as well as capture the proton therapy treatment effects (Fig. 1d, see Methods).

### Nuclear chromatin phenotypes of PBMCs distinguish control and tumor populations

**Fig. 2:**
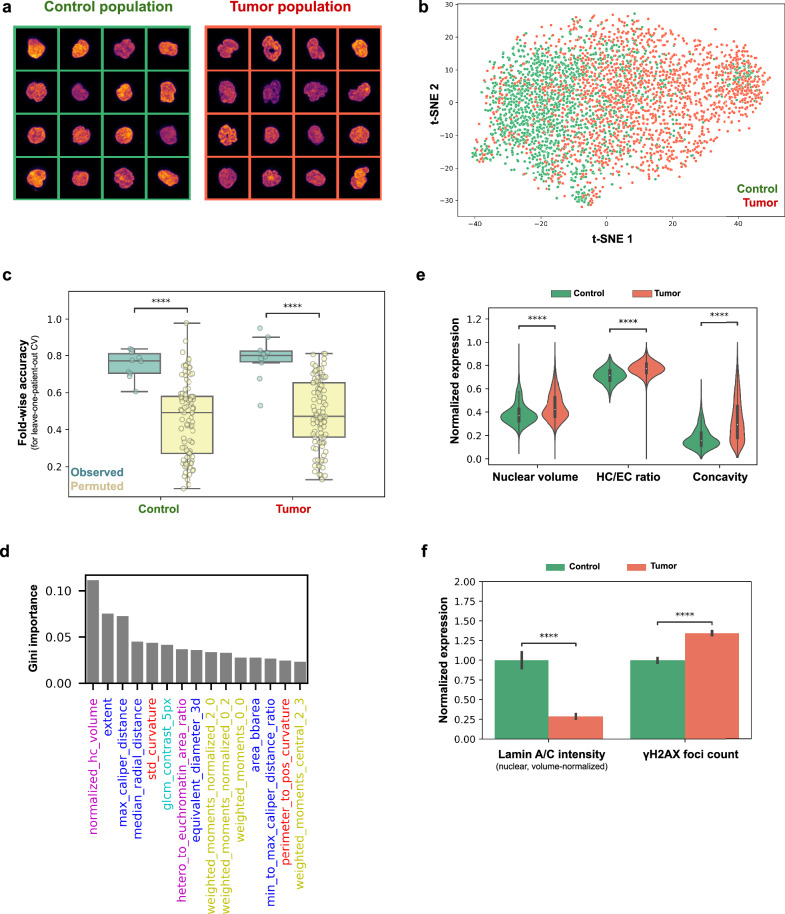
PBMCs of control and pan-tumor patients show distinct nuclear chromatin phenotypes. **a** Visualization of 16 representative single-nuclei images of the control (left) and the pan-tumor population (right). Maximum projections of the 3D single-nuclei are shown where the coloring encodes the observed DNA intensity (warmer is higher). Each image corresponds to 13.5 × 13.5 microns. **b** T-distributed stochastic neighbor embedding (tSNE) of the PBMCs of the control (green) and the pan-tumor population (red). The embedding is calculated using the derived chrometric profiles for each PBMC (see Methods). **c** Boxplot showing the performance of a RandomForest classifier (RFC) (in light green) against a random baseline (light yellow) for identifying PBMCs of the control and the pan-tumor population using their respective chrometric features. The performance is evaluated using leave-one patient-out cross-validation and the random baseline is obtained by permuting (10 times) the condition labels of the PBMCs (see Methods). *P*-values < 10^-4^, two-sided Wilcoxon rank-sum test. **d** Importance of the chrometric features as determined by the RandomForest classifier to distinguish between PBMCs from the control and the pan-tumor population. The chrometric features are colored by their corresponding category: Features related to the DNA intensity distribution are colored in green, Image moment features in yellow, HC/EC related features in pink and features associated to the shape of the nuclear boundary in red. The 15 features with the highest Gini importance are shown. **e** Violin plot showing the range-normalized expression of the three most differentially expressed chrometric features of the control (green) and pan-tumor population (red). *P*-values < 10^-63^, two-sided Welch’s *t*-test. **f** Bar plot showing the average expression of two protein-related features of PBMCs in the control (green) and the pan-tumor (red) population. The expression was normalized by dividing it by the mean of the control condition. Error bars correspond to one standard deviation. *P*-values < 10^-36^, two-sided Welch’s *t*-test.

To establish the potential of using the 3D chromatin organization of PBMCs as a read-out of the systemic health/disease state of an individual/patient, we first obtained PBMCs from healthy controls (*n* = 10) and patients with tumors (*n* = 10, see Supplementary Table 1). The chromatin state of the PBMCs, was characterized using the chrometric features extracted from corresponding DNA images of the PBMCs (Fig. 1C, see Methods). We selected a balanced subset of PBMCs from the tumor patients (*n* = 1850) and the healthy donors (*n* = 1850) and observed that the chromatin states of the PBMCs of the healthy controls and the tumor patients showed prominent differences (Fig. 2a). For instance the nuclei of the PBMCs were often seen to be fragmented in the tumor population. The different chromatin states of the PBMCs from the control and tumor population were also captured by the extracted chrometric features as shown in a t-distributed neighbor embedding (tSNE) plot (Fig. 2b). Importantly, the PBMCs were not seen to cluster by their individual donors and thus no evidence for batch effects was observed (Supplementary Fig. 2a).

We next quantified the dissimilarity of the chromatin states of the PBMCs between the control and the tumor population by evaluating a random forest classifier (RFC) using the chrometric features as input trained to distinguish between PBMCs from the two populations in a leave-one-patient-out cross-validation scheme (see Methods). The RFC achieved an average classification accuracy of 0.74 ( + /− 0.10) and an average specificity of 0.76 ( + /− 0.12) and a sensitivity of 0.72 ( + /− 0.08) (Fig. 2c). As expected, an ablation study showed that the classification accuracy degraded if fewer nuclei or fewer patients were used to train the model (Supplementary Fig. 2d, e). However, even when trained solely on PBMCs of as little as one randomly selected control and tumor patient, the model was able to predict if a given PBMC comes from a held-out patient with or without a tumor (Supplementary Fig. 2d, see Methods) with an average accuracy of 0.70 ( + /− 0.03). Importantly, these metrics describe the performance of the RFC on a single-cell level, i.e. to predict for a single PBMC if it comes from a tumor or a control patient. When aggregating the predictions for all PBMCs on a patient-level and classifying each patient based on which condition was predicted most frequently for PBMCs of the respective patient, the RFC was seen to achieve perfect classification accuracy, i.e. for all patients the majority of the corresponding PBMCs are classified correctly (Supplementary Fig. 2f).

The classifier was seen to especially use chrometric features measuring the nuclear volume, shape and heterochromatin content of PBMCs to distinguish between the control and tumor populations (Fig. 2d). A statistical screen validated that these features were most differentially expressed (Supplementary Fig. 2e, see Methods). Interestingly, the large-scale differences between the control and the tumor population PBMCs was also well reflected using a linear discriminant analysis (LDA) (Supplementary Fig. 2d). Previous studies have implicated the connection between nuclear Lamin and DNA damage for the chromatin organization of immune cells^42,43^. Using our pipeline, we found that Lamin A/C is decreased in PBMCs of the tumor population compared to the control population (Fig. 2F, see Methods). We also observed significantly higher DNA damage contents in the PBMCs of the tumor population (Fig. 2g), likely caused by their exposure to the tumor secretome as previously reported^44^. Interestingly, preceding chemotherapy was not seen to be significantly reflected in the PBMCs of our study population (Supplementary Fig. 2b, c).

### Nuclear chromatin phenotypes of PBMCs identify different tumor groups

**Fig. 3:**
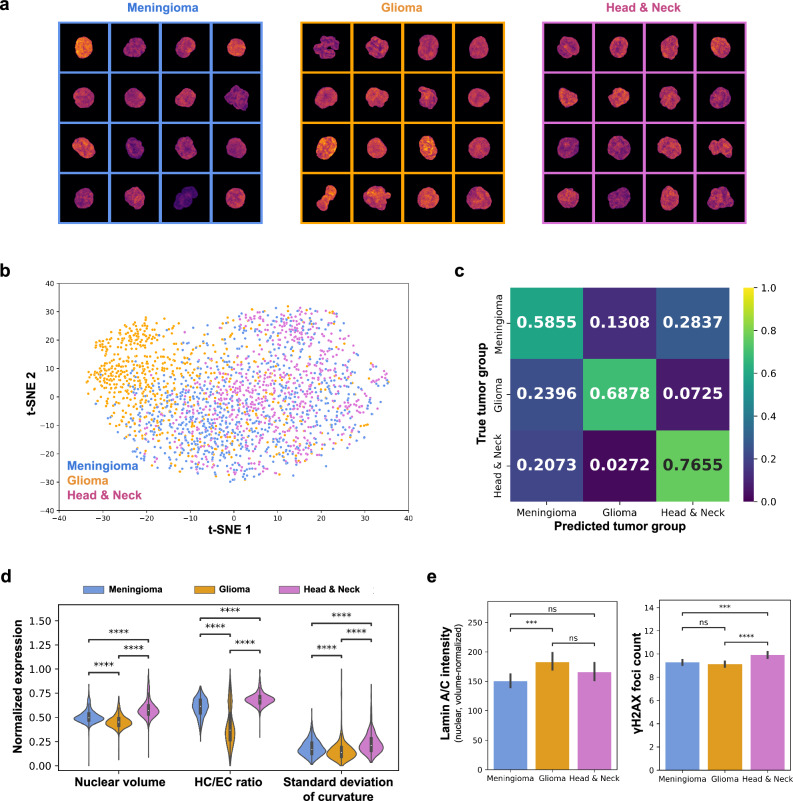
Nuclear chromatin organization separates tumor group populations. **a** Visualization of 16 representative single-nuclei images of PBMCs of meningioma, glioma and head and neck tumor patients (left to right) obtained from the study population prior to proton therapy. Maximum projections of the 3D single-nuclei are shown where the coloring encodes the observed DNA intensity (warmer is higher). Each image corresponds to 13.5 × 13.5 microns. **b** Visualization of the chrometric profiles of different PBMC populations represented by their chrometric profiles using a tSNE plot. Points representing individual PBMCs (*n* = 2316) are colored according to the tumor group of the patients (*n* = 20) they originate from. **c** Average of the row-normalized confusion matrices for a RFC evaluated in a leave-one-patient-out cross-validation scheme on the task of classifying the respective tumor group label of PBMCs given their chrometric profiles (see Methods). The classifier achieves an average accuracy of 0.70 ( + /− 0.29). **d** Violin plot showing the range-normalized expression of the three most differentially expressed chrometric features between the meningioma (blue), glioma (orange) and head and neck tumor group population (pink). *P*-values < 10^-11^, two-sided Welch’s *t*-test. **e** Bar plots showing the average expression of two protein-related features of PBMCs in the three tumor group PBMC populations. Error bar corresponds to one standard deviation. *P*-values are coded as follows: *p*-value > 0.05 (ns), *p*-value < 0.05 (*), *p*-value < 0.01(**), *p*-value < 0.001 (***), *p*-value < 0.0001(****), two-sided Welch’s *t*-test.

The previous findings show that the chromatin organization of PBMCs is altered when exposed to the tumor secretome. The composition of the secretome signals depends on the tumor state and tumor type^8,45^ and thus these differences should be reflected in the chromatin states of PBMCs. To test this hypothesis, we analyzed blood samples of 8 of 10 glioma, 4 of 10 meningioma and 8 of 10 head and neck tumor patients (with sufficient cells) using our presented pipeline (Fig. 1a, Supplementary Table 2–4, see Methods). While visually the PBMCs from the different tumor groups looked similar (Fig. 3a), the chrometric features showed significant differences of the chromatin states in the different tumor groups. This is highlighted by a tSNE visualization where the PBMCs are clustered by tumor group (Fig. 3b). No evidence for technical batch effects was observed (Supplementary Fig. 3a).

We next quantitatively assessed the separability of the PBMCs of the different tumor populations by their corresponding chromatin states. To this end, we evaluated the performance of a RFC to distinguish between PBMCs from the different tumor populations in a similar leave-one-patient-out cross-validation scheme (see Methods). The model achieved an average classification accuracy of 0.70 ( + /− 0.29) and identified PBMCs from head and neck tumor patients with an accuracy of 0.77. For Meningioma and glioma patients, 59% respectively 69% of all PBMCs were correctly classified by the RFC. Interestingly, the RFC was seen to frequently confuse PBMCs from meningioma and glioma patients, suggesting similar chromatin states of the PBMCs of these two brain tumor groups. This is further reflected in the overlap of the respective PBMC distributions in a LDA plot (see Supplementary Fig. 3b). Importantly, we find that for 17 of the 20 tumor patients (i.e. 85% of all patients) the majority of their corresponding PBMCs are classified correctly by the RFC classifier, i.e. our model achieves a classification accuracy of 85% on a patient-level.

We observed that the nuclear volume, heterochromatin content, concavity and the curvature of the nuclear boundary differed significantly between the PBMCs of the three tumor group populations (Fig. 3d). In alignment with our previous results, we observed PBMCs from head and neck tumor patients to be highly dissimilar compared to PBMCs of the other two tumor groups. For instance, their nuclear and heterochromatin volume in the former group was seen to be significantly increased (Fig. 3e), as well as the number of ɣH2AX foci. Interestingly, the Lamin A/C expression levels showed little differences between the different tumor group populations (Fig. 3f).

### Chromatin organization of PBMCs reflects treatment effects of proton therapy

**Fig. 4:**
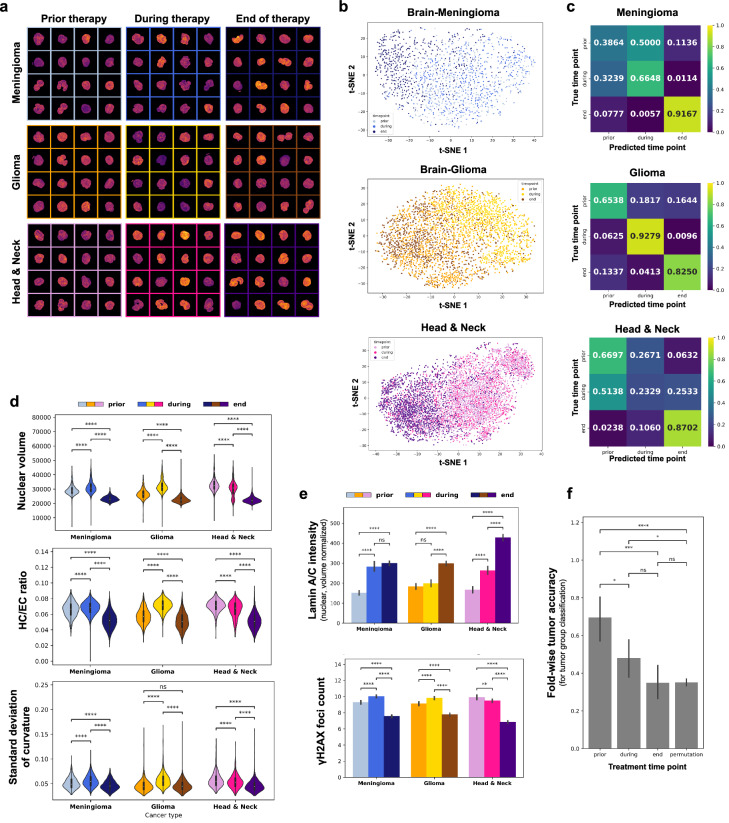
Chromatin organization of PBMCs reflects treatment effect of proton therapy. **a** Visualization of 16 representative single-nuclei images of PBMCs of meningioma, glioma and head and neck tumor patients (top to bottom) obtained from the study population prior to, during and at the end of proton therapy (left to right). Maximum projections of the 3D single-nuclei are shown where the coloring encodes the observed DNA intensity (warmer is higher). Each image corresponds to 13.5 × 13.5 microns. **b** Visualization of the chrometric profiles of the PBMCs of the meningioma (top, n=1584), glioma (center, n=3120) and head and neck tumor population (bottom, n=6312) using a tSNE plot. Each point represents a single PBMC which is colored accordingly to separate PBMCs from samples obtained prior to, during and at the end of proton therapy. **c** Average of the row-normalized confusion matrices corresponding to the performance of a RFC trained on the task to distinguish between PBMCs of the different treatment timepoints given their chrometric profiles and evaluated in a leave-one-patient-out cross-validation scheme. Confusion matrices are given for each tumor group; meningioma (top), glioma (center) and head and neck tumors (bottom). **d** Violin plots showing the expression of three chrometric features that are most differentially expressed in the PBMC populations corresponding to the different treatment time points (n=3132 in total for each tumor group). *P*-values are coded as defined in Fig. 3e, two-sided Welch’s *t*-test. **e** Bar plots showing the average expression of two protein-related features of PBMCs of the different tumor group and treatment time point populations (n=3132 in total for each tumor group). Error bar corresponds to one standard deviation. *P*-values are coded as defined in Fig. 3e, two-sided Welch’s *t*-test. **f** Bar plot visualizing the average leave-one-patient-out cross-validated classification accuracy of a RFC used to distinguish between the PBMC populations of the different tumor groups given their respective chrometric profiles (n=3132 in total for each tumor group). The RFC is trained and evaluated individually for the PBMC populations of the same patients for each treatment time point and compared to a random baseline, which is established via permuting (10 times) the tumor group labels. *P*-values are coded as defined in Fig. 3e, two-sided Wilcoxon rank-sum test.

The previous results highlight the sensitivity of PBMCs to the tumor secretome signals and validate that the presence of such signals is reflected in the PBMCs’ chromatin states. During proton therapy, it is expected that the signal concentration will reduce if the therapy is effective against the tumor. To assess if the treatment effect is also reflected in the chromatin states of the PBMCs, we next used our pipeline to analyze blood samples of the same 30 tumor patients as before; obtained not only prior to but also during and at the end of proton therapy (Fig. 4a, see Methods).

The chromatin states of the PBMCs seemed to primarily differ between treatment time points as seen in a tSNE visualization (Fig. 4b, Supplementary Fig. 4a). Concordantly, a RFC could accurately distinguish between PBMCs of the different treatment time points across all tumor groups, i.e. with classification accuracies of 0.73 ( + /− 0.10) for meningioma, 0.82 ( + /− 0.12) for glioma and 0.53 ( + /− 0.14) for head and neck tumor patients’ PBMCs (Fig. 4c). However, the similarity of the PBMC populations from the different treatment time points were observed to vary between tumor groups. For instance, the RFC classifier was seen to frequently confuse PBMCs from the meningioma and head and neck tumor patients obtained prior for PBMCs obtained during the proton therapy treatment implying a delayed reflection of the treatment effect in the chromatin states of the PBMCs. In contrast, the PBMCs of the glioma patients were accurately distinguished by the classifier for all different treatment time points, which indicates an immediate reflection of the treatment effect in their chromatin states.

We found the nuclear volume, heterochromatin content and the variability of the nuclear curvature to be most differential between the individual treatment time points for the different tumor populations (Supplementary Fig. 4b). Importantly, these features were also found to be increased in PBMCs of the tumor population when compared to the PBMCs of the healthy donors. We observed a reduction of all those chrometric features towards the end of the proton therapy across all tumor groups (Fig. 4d). Additionally, we found a recovery of the nuclear Lamin expression and a reduction of the DNA damage content over the course of the treatments (Fig. 4e). Similar as for the chrometric features, these changes were in opposite direction to the differences between PBMCs of healthy and pan-tumor population (Fig. 2).We further observed that the tumor-specific signature of the PBMCs is sequentially lost during proton therapy. This was seen when training a RFC to discriminate between the different tumor groups on data from each treatment time point individually. The RFC identified the corresponding tumor group for a given PBMC with an accuracy of 70% for PBMCs obtained prior proton therapy, but could no longer predict the different tumor groups significantly better than by chance for PBMCs obtained at the end of the proton therapy (Fig. 4f). Jointly, these findings suggest the reflection of the treatment effect over the course of proton therapy in the chromatin states of PBMCs in all assessed tumor group populations.

### Reflection of cell-type-specific effects of proton therapy in the chromatin organization of PBMCs in different tumor groups

PBMCs consist of a variety of different lymphocytes with distinct cellular functions^46^. We next assessed if our results could be due to the changing abundances of these subpopulations. To this end, we used our pipeline to assess the abundance of PBMCs positive/negative for CD4, CD8 and CD3 in the blood samples from the 30 tumor patients over the course of proton therapy (see Methods). We first observed a decreased abundance of CD4 + CD8- (CD4+ positive) cells in all tumor groups prior to the proton therapy treatment. In contrast, the number of CD4-CD8+ (CD8 + ) cytotoxic cells was seen to be generally increased in the PBMCs of the tumor patients compared to the PBMC population of the healthy donors. Interestingly, the abundance of the CD8+ cells was seen to further increase over the course of the proton therapy treatment in the glioma population.

**Fig. 5:**
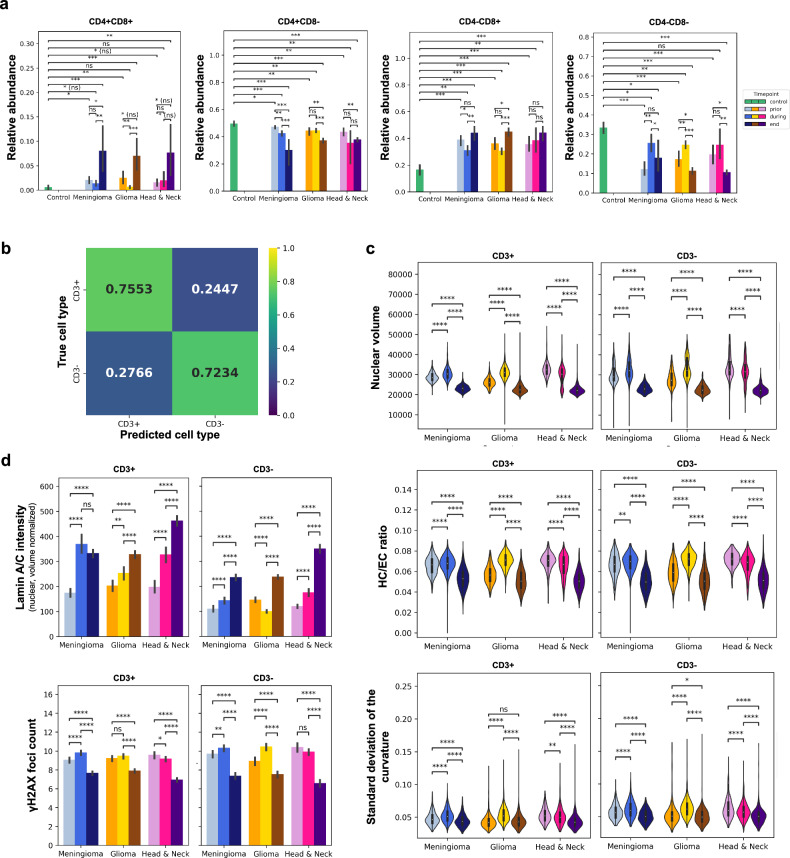
Reflection of cell type-specific effects of proton therapy in the chromatin organization of PBMCs. **a** Bar plots showing the relative abundance of different PBMC subsets in the control and the three tumor group populations over the course of proton therapy. The average across the patient samples per condition and treatment time point are (with sufficient cells; n≥6 each) shown and the error bar corresponds to one standard deviation. *P*-values are coded as defined in Fig. 3e, two-sided Welch’s *t*-test. **b** Average of the row-normalized confusion matrices reflecting the performance of a RFC evaluated in a leave-one-patient out cross-validation scheme and trained to distinguish between CD3+ (T) cells and CD3- (Non-T) cells given their chrometric features across all disease/health and treatment time point conditions. **c** Violin plots showing the expression of the three chrometric markers from Fig. 4d separately for CD3 + /− PBMCs in the PBMC populations of the different tumor groups and across the different treatment time points. *P*-values are coded as defined in Fig. 3e., two-sided Welch’s *t*-test. **d** Bar plots showing the average expression of two protein-related features of PBMCs separately for CD3 + /− PBMCs of the different tumor group and treatment time point PBMC populations. Error bar corresponds to one standard deviation. *P*-values are coded as defined in Fig. 3e, two-sided Welch’s *t*-test.

Moreover, CD4-CD8- cells were observed to be less and CD4 + CD8+ cells more abundant in all tumor groups compared to the control population. The abundance of the CD4 + CD8+ cells was seen to increase over the course of proton therapy. In contrast, the abundance of the other subtypes, including CD3 + /− cells, showed small variations across the different treatment time points (Fig. 5a), potentially due to the large patient-specific variability (Supplementary Fig. 5a).

To assess if the previously observed changes of the chromatin states of the PBMCs during proton therapy were potentially confounded by the reported changes of the abundance of individual cell types, we validated that CD3 + /− cells showed different chromatin states. To this end, we showed that a RFC could discriminate between CD3 + /− PBMCs with an accuracy of 0.74 ( + /− 0.03), which is significantly better than random chance (Fig. 5b, Supplementary Fig. 5b, c, see Methods). However, the previously described treatment effects were similarly reflected in the chromatin organization, their Lamin A/C and ɣH2AX profiles of CD3+ and CD3− PBMCs (Fig. 5c, d). Furthermore, we found the classifier trained to distinguish between the three different tumor types to achieve a slightly lower balanced accuracy when being trained only on CD3+ respectively CD3- cells achieving a balanced accuracy of 0.67 ( + /− 0.30) and 0.61 ( + /− 0.32) respectively (Supplementary Fig. 5b, c). Recalling the results of our ablation study, this reduction is likely due to the reduced number of PBMCs available to train the models when sub-setting them to CD3+ or CD3- cells (Supplementary Fig. 3e). More importantly, the previously described treatment effects were also similarly reflected in the chromatin organization, their Lamin A/C and ɣH2AX profiles of CD3+ and CD3- of CD3 + /− PBMCs (Fig. 5d, e). Jointly these results suggest that, while there exist distinct chromatin states of different cell types of PBMCs, the previously identified alterations of the chromatin states of PBMCs are not explained by such cell-type specific differences, but all PBMC subsets show a similar reflection of changes of the concentration and composition of tumor secretome signals in their chromatin organization.

## Discussion

The development of reliable prognostic biomarkers for patients with tumors is critical for personalized therapy, enhanced diagnostics and monitoring treatment effects. Primary tumors secrete a range of signaling molecules into their tumor microenvironment. While the exact composition of the secretome is largely unknown, related research has identified of insulin-like and opioid growth factors or proteins such as ADAM9, cathepsin B and neuropilin-1 as key components of the secretome of head and neck tumor or glioblastoma cells^47,48^. Circulating PBMCs respond to such tumor secretome signals with functional and phenotypic changes, either mounting an immune response or providing aid for cancer progression and metastasis^49,50^. Consequently, liquid biopsies assessing the DNA methylation, miRNA, transcriptional and proteomic profiles of blood cells (e.g. PBMCs), as well as alterations of their specific subpopulations, have become an active area of research^13,20,35,36^. Since the chromatin integrates the functional outputs of PBMCs, we hypothesized that the alterations of the chromatin as a response to the tumor secretome could serve as novel prognostic biomarkers. Towards this, we developed a chromatin imaging and AI-based pipeline that identifies chromatin biomarkers, which characterize the differences between healthy individuals and patients with tumors and provide read-outs for advanced anti-tumor therapy settings. In contrast to single molecular biomarkers or sequencing-based methods, our approach offers a streamlined and resource-efficient means of biomarker detection, catering to the distinct molecular marker expression profiles exhibited by various cancer types for diagnostic purposes^51–53^. The comprehensive high-dimensional representation of chromatin, encompassing its integrative functionality, furnishes a read-out that goes beyond mere identification of cancer presence. This capacity extends to the identification of diverse cancer types and the capture of tumor characteristic alterations during the course of treatment.

Our pipeline robustly detected alterations of the chromatin organization of PBMCs from healthy donors and tumor patients treated with proton therapy. We found significant differences of chrometric features such as the nuclear volume, concavity and curvature which have been previously linked to activation of PBMCs^54–56^. In further agreement, previous studies revealed MAPK, NK-kB dependent changes of chromatin structure and the accompanying gene expression as response to tumor secretome signals^33,57,58^.

We validated that these chromatin biomarkers could discriminate between PBMCs of meningioma, glioma patients and head and neck tumor patients with an average accuracy of 70%. Importantly, the results of the ablation study imply that the performance of our RFC to distinguish between PBMCs of control and patients with different tumor types could further increase by expanding the study population to a larger set of patients (Supplementary Figs. 2d and 3d) and/or increasing the number of PBMCs sampled for each patient (Supplementary Fig. 2e and 3e). Our analyses further showed that PBMCs from glioma and meningioma patients show similar chromatin states which are highly dissimilar from head and neck tumor patients. This aligns with previous research which showed dissimilar secretome compositions of brain tumors such as meningioma and glioma compared to head and neck tumors^59,60^.

To assess the efficacy of proton therapy outcomes, we applied our pipeline to assess differences of the chrometric features of PBMCs obtained at different treatment time points during proton therapy of meningioma, glioma and head and neck tumor patients. The treatment effects were differently reflected in the PBMC populations of the three different tumor groups, suggesting tumor-specific treatment responses. These should be further assessed in a large clinical trial due to their potential implications for proton therapy administered to patients with tumors. Overall, our results show that over the course of proton therapy, PBMCs of tumor patients showed a partial recovery of important chrometric features, such as the nuclear volume, HC/EC ratio, and concavity, approaching the levels of the healthy donor population. Concordantly, we observed a sequential loss of the tumor-specific signature in the chromatin states of the PBMCs over the course of the treatments. This loss of separability could be due to the reduced tumor secretome signals sensed by the PBMCs as a result of proton therapy.

To complement our analyses of the chromatin alterations, we studied nuclear organization (Lamin A/C levels) and DNA damage content (ɣH2AX levels) in PBMCs over the course of proton therapy. The Lamin A/C levels were found to be decreased in PBMCs of patients with tumors compared to PBMCs from healthy donors, suggesting that the nuclear architecture may be disrupted in patients in the former group. Interestingly, Lamin A/C levels were significantly increased over the course of proton therapy, suggesting an interplay between immune response and proton therapy effects. Furthermore, we observed the DNA damage content to be significantly increased in PBMCs of patients with tumors, suggesting that the tumor secretome leads to an impairment of the DNA repair mechanisms. At the end of proton therapy, PBMCs showed reduced DNA damage, suggesting the efficacy of proton therapy treatments. In agreement with previous studies^61–63^, we found the CD4+ population to be reduced, whereas the CD8 + PBMC population was seen to be increased in the tumor population. Interestingly, the CD8+ PBMC population was also observed to be increased during proton therapy in glioma patients. This highlights the effects of proton therapy on cytotoxic T cell responses, which is consistent with previous chemotherapy results^64^. These findings also indicate the potential of our imaging and AI pipeline to further benefit from separation of the PBMCs by cell type using e.g. cell sorting approaches and study the cell-type specific alterations of the chromatin organization of PBMCs in response to the tumor signal, potentially yielding a better accuracy for diagnoses. While our preliminary analyses using fluorescent markers to subset the PBMCs into CD3+ and CD3- cells did not show an improvement of the classification accuracy of our models, we hypothesize that a more fine-grained distinction between different PBMC subsets might prove useful for tumor type diagnoses and should be addressed in future work.

In summary, we have developed an imaging and AI-based approach to identify chromatin biomarkers in PBMCs using liquid biopsies. The identified chromatin biomarkers can accurately discriminate between PBMCs of healthy donors and patients with various tumors, as well as from different proton therapy treatment time points. Chromatin organization features and nuclear morphology features, such as the nuclear volume and concavity, were found to accurately distinguish PBMCs from healthy donors and patients as well as to separate different tumor groups. We believe that the presented approach using simple imaging techniques and machine learning to identify chromatin biomarkers has wide-ranging applications in disease diagnostics and therapy evaluation.

## Methods

### Study population

A total of 10 healthy and 10 patients with tumors were used to first assess the sensitivity of chromatin biomarkers to discriminate between healthy and tumor patients (see Supplementary Table 1). We then used a total of 30 tumor patients, i.e. glioma, meningioma and head and neck tumor patients (*n* = 10 each), to study the reflection tumor-specific differences and the proton therapy treatment effects in the chromatin organization of PBMCs (see Fig. 1a, Supplementary Table 2).

### Study protocol

Blood samples of patients undergoing proton therapy were collected at three different time points, i.e. the first sample before proton therapy, the second sample in the middle of the therapy and the final sample within the last week of the proton therapy. Control blood samples were obtained from the donors who experienced no relevant previous medical history of tumors or chronic disorders. 10 ml of blood from both adult healthy donors and the tumor patients as well as 3–10 ml of blood from pediatric patients were collected in EDTA coated tubes. The detailed study protocol was approved by the Ethics Committee of Switzerland with the approval number EKNZ 2021-00481 and the Center of Proton Therapy of the Paul Scherrer Institute. The protocol is in compliance with all relevant ethical regulations including those defined in the Declaration of Helsinki. As described in the detailed study protocol, which is available from the aforementioned approval number, all samples from healthy donors and patients were collected with informed written consent.

### Peripheral Blood Mononuclear Cell (PBMC) isolation

PBMCs were isolated by following the established protocol for density gradient centrifugation. In brief, phosphate-buffered saline (PBS) + 2% fetal bovine serum (FBS) was used to dilute the blood samples at 1:1 ratio, and then the mixture was added to the Lymphoprep density gradient medium (STEMCELL Technologies, catalog no. 07811) in a SepMate tube from STEMCELL Technologies (SepMate^TM^-50). The tubes were centrifuged at 1200 g for 20 min at room temperature to segregate the PBMCs, which were then enriched in the top layer. The PBMCs were decanted into new tubes, washed two times with cold PBS + 2% FBS. 3 × 10^6^ cells were fixed with ice cold 4% Paraformaldehyde (PFA) for 15 min and used for immunostaining and microscopy. Finally, remaining PBMCs were stored at liquid nitrogen (-135 °C) for future use.

### Immunostaining and microscopy

To prepare PBMCs for fluorescence microscopy, glass coverslips are coated with charged poly-L-lysine for 3 to 4 hr at room temperature to induce cellular attachment. After PFA fixation, 1 ×10^6^ cells were plated onto the coated coverslips for 3 hr and washed with PBS for 3 times. Cells were permeabilized with 0.1% (w/v) Triton- X100 for 10 min and washed once again with PBS for 5 min. Further, cells were blocked with 1% BSA for 30 min and washed one more time with PBS. Next, antibodies were used for immunostaining of PBMCs: monoclonal rabbit anti-γ-H2AX from Cell Signaling Technologies (catalog no. 2577 S), monoclonal mouse anti-Lamin A/C from Abcam (catalog no. ab8980), monoclonal rat anti-CD3 from Abcam (catalog no. ab11089), recombinant Alexa Fluor 647 anti-CD8 alpha antibody from Abcam (catalog no. ab196193), monoclonal mouse anti-CD4 from R & D systems (catalog no. MAB379), recombinant rabbit anti-CD16 from Abcam (catalog no. ab246222). The following secondary antibodies were used: Alexa Fluor 488 anti-mouse Ab (catalog no. A32723), Alexa Fluor 555 anti-rabbit Ab (catalog no. A32794) and Alexa Fluor 647 anti-rat (catalog no. 32728). Confocal microscopy was carried out on a Leica Stellaris (inverted DMI8) running the LAS X software. PBMCs slides were imaged with 63X /1.4 NA oil immersion objective.

### Image processing and feature extraction

#### Image data preprocessing

Raw multi-channel images of PBMCs fluorescently labeled with DAPI, the functional (γH2AX, Lamin A/C) and cell-type markers (CD3, CD4, CD8) with a resolution of 0.09 µm in the x-y plane and 0.5 µm in z-direction were converted into TIFF images using ImageJ (version 1.53c)^65^. The DAPI images were range-normalized to account for the observed variability of the explored ranges of the intensity spectra of the individual images.

#### Nuclei segmentation

Single-nuclei masks and cellular masks of the PBMCs were identified in a multi-step procedure. First, we obtained nuclear masks in 2D. To this end, we computed 2D projections of the DAPI images via max-z projection. Noise was removed from the max-z projected images using a median filter. To enhance the contrast of the DAPI images, the intensities were gamma-adjusted with γ = 0.7. 2D Nuclei masks were then obtained via automatic thresholding using Otsu’s method^65^ and identifying connected components in the resulting binary images. Segmentation artifacts were removed using a size filter that only kept segmented objects with an area of at least 800px (6.48µm^2^) and at most 5000px (40.5 µm^2^). Note that this step also removed all nuclei that were touching other nuclei in 2D. Objects touching the boundary of the images were also excluded.

Next, we used the resulting 2D segmentation masks to guide the 3D nuclear segmentation. In particular, we expanded the bounding boxes of the 2D nuclear masks by roughly 1 micron (12px) and obtained 3D crops of the z-stack (3D) multi-channel images. To this end, we cropped the z-stacks at the location of the expanded bounding boxes in the x-y plane. The expansion of the bounding boxes was done to account for the fact that most of the proteins we have stained for are only expressed on the nuclear and cellular periphery. Background signal was removed from the resulting 3D multi-channel nuclear crops by setting the background intensities to 0. Thereby, the background was identified as the region outside of the convex hulls of the 2D nuclear masks after dilating the masks 12 times with a cross-shaped structuring element of 3×3 dimensions. As before the dilation was done to obtain an approximate mask that contains not only the nucleus but the complete cell.

We perform a similar approach to remove additional background signals from the DAPI channel of the crop images. In particular, we identify an approximate single-nuclei mask in 2D for the resulting crop images. To this end, we again apply a median filter to a 2D max-z projection of the DAPI image of the 3D crops. We then obtain a 2D nuclear mask via thresholding the image using Otsu’s method^66^. Any holes in the obtained binary masks are filled. We then set all regions outside of the 2D nuclear masks to 0 of the 3D DAPI crop images. Nuclear masks in 3D are obtained using the Chan-Vese algorithm^67^ applied to the 3D DAPI crop images. The algorithm is run for a maximum of 300 epochs with the hyper parameters (λ_1_, λ_2_) = (1, 2). Finally, 3D segmentation artifacts were removed by filtering out objects with a size of <400 voxels (1.62µm^3^). Finally, we removed cut off or overlapping nuclei in 3D by only keeping nuclei with a height of 2.5-10 µm which was measured along the z-axis. Supplementary Figure 1b provides a visual representation of the 3D nuclear segmentation quality.

#### Cell segmentation

Approximate cellular masks are obtained in 2D and 3D by expanding the boundaries of the respective nuclear masks by 12 pixels in x-y and 2 pixels in z-direction which corresponds to approximately one micron in each direction. This follows an approach recently proposed in ref. ^40^ and exploits the approximate ball-like shapes of the PBMCs.

#### Cell feature extraction

Using the 2D and 3D nuclear masks we obtain a number of features from the DAPI images that describe the nuclear morphology and chromatin organization of the cells using an adaptation of the chrometric python package^40^. Those features include i.e. the volume of the nucleus, its concavity or various texture features. Additionally, the expression of the functional and cell-type marker proteins were quantified by descriptive statistics of the intensity distribution of the corresponding images immunofluorescently stained for these markers. A complete list of all features is available in our publicly available Github repository (see Data and Code availability section) Note that the features are sensitive to PBMC storage, sample preparation conditions and different microscopy imaging settings and therefore appropriate experimental conditions have to be used for reproducible feature extraction.

#### Detection of γH2AX foci

γH2AX foci were identified for each cell from the microscopy images using the 3D nuclear masks. In particular, the single-cell images and the corresponding nuclear masks were projected to 2D using max-z projection. Next, images were range- normalized. We obtained a binary mask for the γH2AX foci by identifying the regions of the image with an intensity at least 2.5 standard deviations above the average intensity. False positive foci were removed by imposing a size filter of 4 pixels, i.e. 0.032μm^2^. To separate overlapping foci, we used the Watershed algorithm with the seeds set to the local intensity maxima of the single-nuclei image. Finally, segmentation artifacts were removed by once again removing objects <4 pixels. Supplementary Figure 1C provides a visual representation of the quality of the described pipeline to detect γH2AX foci.

#### Cell type identification

Cells that stained positive for a specific cell-type marker were identified using the corresponding single-cell images showing the immunofluorescent labeling for a given cell type marker. In particular, we computed the sum of the intensity of the corresponding images within the nuclear mask as a proxy of the expression of the corresponding cell type marker in the given cell. Thereby, we used the max-z projected 2D instances of the 3D nuclear images and the corresponding 2D nuclear mask. The computed sum of the intensities were then normalized by the projected cell area computed from the nuclear masks to account for the heterogeneity in the sizes of the cells. Using a two-component Gaussian Mixture model, we identified a threshold for each patient blood sample that distinguishes cells that are positive for a given marker from those that are negative for the marker. Thus, the pipeline finally outputs the cell type labels of the assessed PBMCs. Supplementary Figure 1D shows an example image highlighting the quality of the described cell type detection pipeline for two of the three cell-type markers.

### Analyses of the chromatin states

#### Data set preprocessing

To analyze the chrometric states of PBMCs in the context of the different health, disease and treatment conditions of the donors, we used the previously described the features extracted from the single-cell chromatin images describing the nuclear morphology and chromatin organization of the PBMCs, i.e. the chrometric features described before. Many of the chrometric features are highly correlated with other chrometric features by their definition. For instance, due to the regular shape of PBMCs the nuclear volume and the projected area are highly correlated. For all analyses presented in the paper, we first removed highly correlated chrometric features, i.e. chrometric features correlated with any other feature with an absolute Pearson correlation coefficient of >0.8 were removed. Additionally, the data was randomly subset such that we had an equal number of PBMCs from the control respectively the different treatment and treatment time point conditions. Additionally, the PBMCs of tumor patients were randomly selected such that an equal number of PBMCs was used from each patient. This was done in order to ensure that individual patient effects cannot dominate the analyses.

#### Data sampling

To ensure an equal representation of each tumor patient sample in our conducted analyses, we randomly selected equally sized random subsets of all PBMCs from all patient samples and treatment time points. This ensured that no patient-specific chromatin organizational characteristics dominated our analyses. In particular, we randomly selected 185 PBMCs for each of the 10 patients of the pan-tumor patient population and an equal number of PBMCs from the healthy donors (*n* = 1850 in total) to assess the differences of the chromatin states of PBMCs in the tumor population compared to the healthy control setting. Similarly, we randomly selected 772 PBMCs from each of the three tumor groups that underwent proton therapy to study the differences of the chromatin states between the tumor groups. Thereby, we ensured that within a given tumor group each patient was equally represented at each treatment time point in our analyses with sufficient cells. Finally, we randomly selected at each treatment time point 528 PBMCs from the meningioma patients, 1040 PBMCs from the glioma patients, and 2104 PBMCs from the head and neck tumor patients (n=11016 in total) to study the reflection of the treatment effects of proton therapy in the chromatin organization of PBMCs (Fig. 4c). We applied a similar sampling approach to jointly evaluate the treatment effects of all tumor populations (Fig. 4d–f). Thereby, we ensured that within a given tumor group each patient was equally represented at each treatment time point in our analyses. Finally, to assess the chrometric differences between CD3+ and CD3- we randomly sampled the same number of nuclei from each tumor group at each treatment time point. Next we obtained a random subsample of those nuclei of 3416 CD3+ and 3416 CD3- cells to assess the chrometric differences of these populations. The sizes of all random samples were generally chosen as large as possible while maintaining the equal representation of each tumor patient sample.

#### Batch effect control

We validated that no evidence for batch effect was found by visually assessing the clustering of PBMCs using t-Distributed Stochastic Neighbor Embedding (tSNE) plots. For none of the reported analyses PBMCs were seen to cluster by patient sample (i.e. patient and treatment time point combination) where each patient sample was processed within one batch (Supplementary Figs. 2a, 3a and 4a-c). Thus, no evidence was found suggesting that the potential technical variations confounded our analyses.

#### Random forest classification to assess chrometric differences

To quantitatively assess the separability of the PBMCs with respect to different healthy and disease conditions, or treatment time points, we evaluated the classification accuracy of a random forest classifier^68^ to distinguish between nuclei of the different conditions. The samples that were input to the classification algorithm were the individual PBMCs represented by the chrometric features.

The performance of the random forest classifiers quantifies how different the chromatin states of the PBMCs that were described by the chrometric features are in the respective conditions. We measured the performance using the balanced classification accuracy^69^ evaluated in a stratified leave-one-patient-out cross-validation approach. Briefly, all PBMCs from the considered conditions are separated by the patient sample they come from. The random forest classifier is then trained on the PBMC data of all but one patient and evaluated on the PBMC data of the patient held out during the training. We repeat this procedure such that the PBMCs from each patient were held out exactly once during training of the RFC and then used to evaluate the balanced classification accuracy of the classifier. Finally, we computed the average of the balanced classification accuracy measures obtained from each iteration to derive an estimate of the overall separability of the compared conditions/classes using the chrometric features of the PBMCs.

Using this setup, during training the classifier has no access to any PBMCs of the patient it is later evaluated on and thus cannot use patient-specific characteristics in the chromatin states of PBMCs for prediction. Hence, the setup enables the evaluation of the classification approach to identify the health condition, disease type or treatment time point of an unseen patient based on the chrometric features of corresponding PBMCs, which mimics a realistic diagnostic settings

#### Ablation study

To assess the influence of the number of patients used to train our classifiers that distinguish (a) between PBMCs of control and tumor patients or (b) between PBMCs of patients with three different tumor types, we performed a systematic ablation study. In particular, we evaluated the performance of the RFC classifier on these two classification tasks using the described leave-one-patient-out cross-validation approach. In each iteration of the cross-validation approach the training set consists of PBMCs of all but one patient whose PBMCs are held-out and used as the test set to evaluate the performance of the classifier (see Methods). In our ablation study, we further randomly subsampled the training set such that only PBMCs of k ϵ {1, 2, …, 9} patients from (a) the healthy and control respectively (b) k ϵ {1, 2, 3} each of the three different tumor types were used to train the model. Once trained on that subset of patients, we again evaluated the performance of the classifier on the PBMCs of the one patient in the held-out test set. Since the performance of the classifier also depended on which patients were randomly sampled for training, we repeated the above procedure 10 times; each time obtaining a random subsample of training patients. For each of these 10 repetitions we computed the average leave-one-patient-out cross-validated accuracy measure for our classifier trained on the PBMCs of the k sampled training patients and evaluated on the held-out test patient.

A similar procedure was adopted to evaluate the influence of the number of PBMCs used to represent each patient and to train our classifiers. The classifiers were again evaluated using the leave-one-patient-out cross-validation procedure. At each iteration we randomly subsampled p ϵ {10%, 20%, …, 100%} of the PBMCs used to represent each patient in the training set and then evaluated the performance of the trained classifier on the PBMCs of the held-out test patient. The procedure was repeated 10 times to obtain 10 measurements of the average.

#### Random baseline via label permutation

To establish a baseline equivalent to random chance, the condition labels (e.g. the tumor group or the time point labels) of PBMCs were randomly permuted in the data set. The permutation of the labels destroys any correlation between the chrometric feature and the condition label. The performance of the classifier trained to predict the condition label of the permuted data set thus provided a robust baseline equivalent to random chance.

#### Biomarker identification

Chromatin biomarkers for various conditions are identified using two methods. First, we identified chrometric features that best distinguished between the different conditions, e.g. PBMCs from healthy donors and tumor patients, by assessing their Gini importance^68^. The Gini importance was output by the RFC trained to distinguish the different conditions. Second, we run a large-scale statistical screen by testing for differences of the means of the chrometric features in the different conditions using Welch’s test. As expected both strategies highlighted the same chrometric features.

### Statistical testing

All statistical tests are explicitly mentioned, whenever *p*-values are reported, i.e. either in the main text as part of the figure legends. All reported *p*-values were adjusted for multiple testing using the Benjamini-Hochberg method and adjusted *p*-values < 0.05 were assessed as statistically significant.

## Supplementary information

Supplemental Information

## Data Availability

The imaging data associated with the presented work is publicly available from the PSI Public Data Repository under the 10.16907/b039dc4e-9366-413c-8f34-92ce9110cc14. Any other data is contained in the manuscript. Requests for additional data and correspondence related to any material presented in the manuscript should be addressed to GV Shivashankar (g.v.shivashankar@hest.ethz.ch).

## Code availability

The developed Python package software used to run the analyses presented in this paper is available at https://github.com/GVS-Lab/chromark. A list of additional software packages our package uses also detailing the exact versions of these packages is provided in the repository.
