# Author Correction: Imaging and AI based chromatin biomarkers for diagnosis and therapy evaluation from liquid biopsies

**DOI:** 10.1038/s41698-025-00894-w

**Published:** 2025-04-24

**Authors:** Kiran Challa, Daniel Paysan, Dominic Leiser, Nadia Sauder, Damien C. Weber, G. V. Shivashankar

**Affiliations:** 1 Mechano-Genomic Group, Division of Biology and Chemistry, Paul-Scherrer Institute, Villigen, Switzerland; 2 Department of Health Sciences and Technology, ETH Zurich, Zurich, Switzerland; 3 Center for Proton Therapy, Paul-Scherrer Institute, Villigen, Switzerland; 4 Department of Radio-Oncology, University Hospital Zurich, Zurich, Switzerland; 5 Department of Radio-Oncology, University of Bern, Bern, Switzerland

**Keywords:** Diagnostic markers, Prognostic markers, Cancer

Correction to: *npj Precision Oncology* 10.1038/s41698-023-00484-8; published online 14 December 2023

In this article, the main text and legends for Supplementary Figures 2-5 had minor errors in image processing which affected the subsequent analysis; the Supplementary Information file has been replaced with the correct version. The original article has been corrected.

