# Supplementary material for: Imaging and AI based chromatin biomarkers for diagnosis and therapy evaluation from liquid biopsies
Source: NPJ Precis Oncol. 2023 Dec 14;7:135. doi: 10.1038/s41698-023-00484-8 (PMC10719365; doi:10.1038/s41698-023-00484-8)
Supplement: Supplementary file 1 — Supplemental Information [file 41698_2023_484_MOESM1_ESM.pdf]

Supplemental Information for

Imaging and AI based Chromatin Biomarkers for Diagnosis and Therapy Evaluation from Liquid Biopsies

Kiran Challa[1,#], Daniel Paysan[1,2,#], Dominic Leiser[3], Nadia Sauder[3], Damien C. Weber[3,4,5,*] , GV Shivashankar[1,2,*]

[1] *Mechano-Genomic Group, Division of Biology and Chemistry, Paul-Scherrer Institute, Villigen, Switzerland*
[2] *Department of Health Sciences and Technology, ETH Zurich, Zurich, Switzerland*
[3] *Center for Proton Therapy, Paul-Scherrer Institute, Villigen Switzerland*
[4] *Department of Radio-Oncology, University Hospital Zurich, Zurich, Switzerland*
[5] *Department of Radio-Oncology, University of Bern, Bern, Switzerland*
[#] Equal contribution
[*] To whom correspondence should be addressed: g.v.shivashankar@hest.ethz.ch

---

This PDF file includes:

- Supplementary Figure 1- 5
- Supplementary Table 1-4

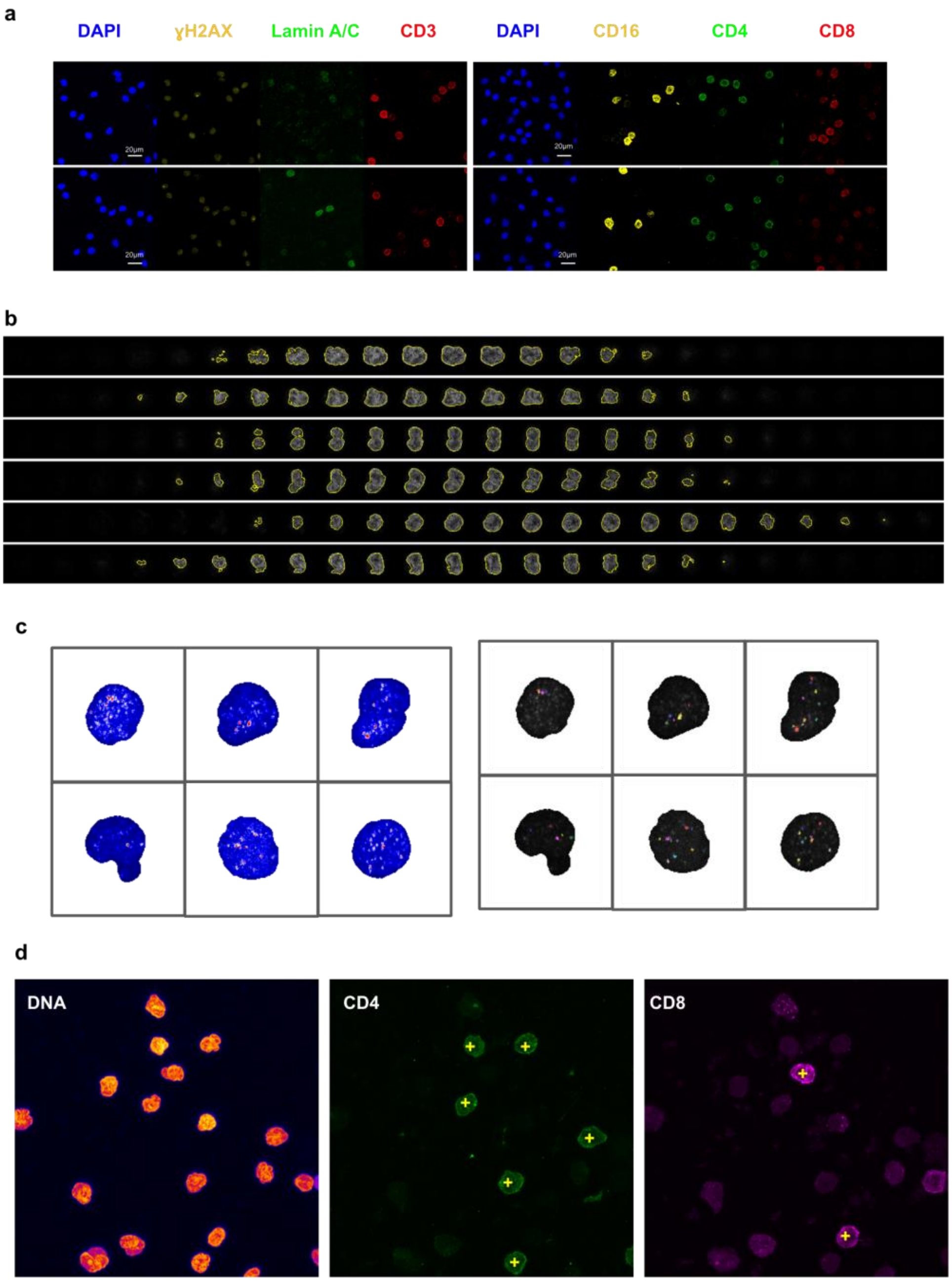

Supplementary Figure 1: Visualization of the computational platform.

- **a.** Overview of representative fluorescently-labeled images (2 images from each staining group) input to our computational pipeline. Maximum z-projections are shown. Scale bar represents 20μm.
- **b.** Visualization of the 3D nuclei segmentation results for 6 randomly chosen nuclei. Each row shows an individual nucleus and each column corresponds to a specific layer of the 3D z-stack image. The segmentation is shown as the yellow outline for each layer.
- **c.** Visualization of the gH2AX foci detection results for six representative nuclei. The detection of gH2AX foci is performed using the maximum-projected single-nuclei images which are shown for 6 randomly chosen nuclei on the right, here warmer colors correspond to higher gH2AX intensity. The left plot shows identified foci and highlights those in color while plotting them against a background that shows the intensity of the single-nuclei image corresponding to the gH2AX channel. Images were range-normalized for better visibility.
- **d.** Visualization of the cell type classification results by showing the max-z projection of a randomly chosen field of view image with the immunofluorescent labels for the DNA, CD4 and CD8 (left to right). Cells labeled positively for CD4 or CD8 by our cell type classification pipeline (see Methods) are marked in the respective images with a yellow plus sign.

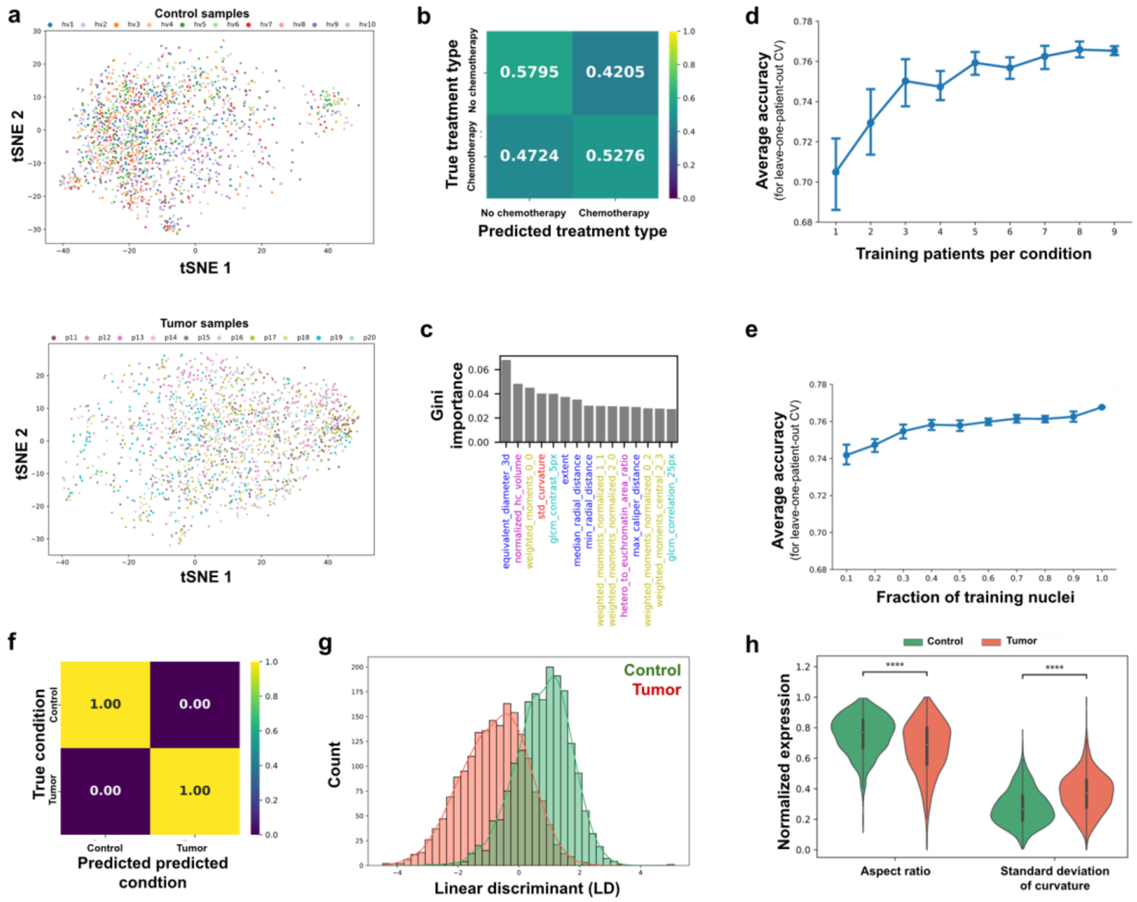

Supplementary Figure 2: Extended analyses of the chromatin phenotypes of PBMCs from the control and the pan-tumor population.

- **a.** Visualization of the inter- and intra-patient variation of the chrometric profiles of the PBMCs in the control (left) and the pan-tumor (right) population using a tSNE plot. PBMCs represented by individual points are colored according to the biological sample they correspond to, i.e. the patient or control sample represented by its unique identifier.
- **b.** Confusion matrix showing the performance of a RFC used to distinguish between patients with and without chemotherapy in the pan-tumor population. The performance of the RFC is evaluated using 5-fold stratified patient-level cross-validation and the row-normalized, average confusion matrix is plotted.
- **c.** Barplot showing the importance of the chrometric features used by RFC to distinguish between tumor patients with/without preceding chemotherapy. The color-coding is done in alignment with Fig. 2 and the top 15 features are shown.
- **d.** Visualization of the average leave-one-patient-out cross-validation accuracy of the random forest classifier (RFC) distinguishing between PBMCs from healthy control and pantumor patients when it is trained on only one, two up to 9 randomly sampled patients per condition.
- **e.** Visualization of the average leave-one-patient-out cross-validation accuracy of the RFC distinguishing between PBMCs from healthy control and pantumor patients when it is trained on only 10%, 20% up to 100% randomly sampled nuclei per patient.
- **f.** Patient-level average of the row-normalized confusion matrices for a RFC evaluated in a leave-one-patient-out cross-validation scheme on the task of classifying the respective condition (i.e. tumor or control label). Predictions on a patient-level by selecting the most frequent prediction label of the RFC applied to the PBMCs of the respective patient.
- **g.** Linear discriminant plot of the control (green) and the pan-tumor PBMC population (red). The LDA achieves an accuracy of 0.75 (+/- 0.13) in a leave-one-patient-out cross-validation scheme.
- **h.** Violin plot showing the range-normalized expression of the two additional chrometric features that are differentially expressed in the PBMC populations of the control (green) and pan-tumor population (red). P-values < 10^{-57}, two-sided Welch's t-tests.

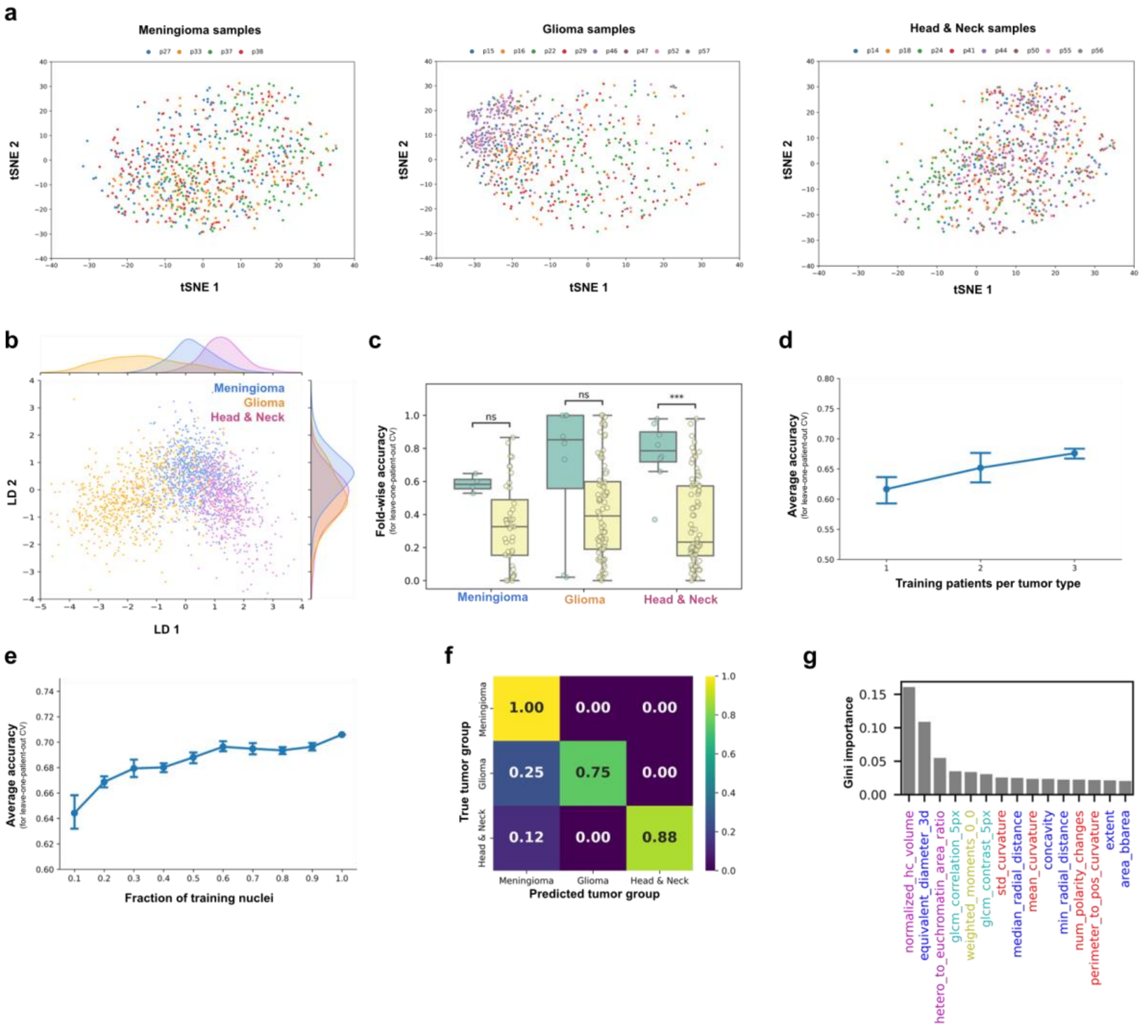

Supplementary Figure 3: Extended analyses of the chromatin phenotypes of PBMCs from three tumor group populations.

- **a.** Visualization of the chrometric profiles of the PBMCs (n=2,316) of the Meningioma (top), Glioma (center) and Head & Neck tumor population (bottom) using a tSNE plot. Each point represents a single PBMC which is colored according to the patient sample it originates from.
- **b.** Linear discriminant plot of the PBMCs of the Meningioma (blue), Glioma (orange) and Head & Neck tumor group population (pink). The LDA achieves an accuracy of 0.67 (+/- 0.29) in a leave-one-patient-out cross-validation scheme on classifying PBMCs with respect to their respective tumor group given the corresponding chrometric profiles.
- **c.** Boxplot showing the performance of a RandomForest classifier (light green) against a random baseline (light yellow) for identifying PBMCs of the three selected tumor group populations using their respective chrometric profiles. The performance is evaluated using leave-one-patient-out cross-validation and the random baseline is obtained by permuting (10 times) the condition labels of the PBMCs (see Methods). P-values are coded as defined in Fig. 3E, two-sided Wilcoxon rank-sum-Wilcoxon test.
- **d.** Visualization of the average leave-one-patient-out cross-validation accuracy of the random forest classifier (RFC) distinguishing between PBMCs from the three tumor group populations patients when it is trained on only one, two or three randomly sampled patients per condition.
- **e.** Visualization of the average leave-one-patient-out cross-validation accuracy of the RFC distinguishing between PBMCs from the three tumor group populations when it is trained on only 10%, 20% up to 100% randomly sampled nuclei per patient.
- **f.** Patient-level average of the row-normalized confusion matrices for a RFC evaluated in a leave-one-patient-out cross-validation scheme on the task of classifying the respective condition (i.e. the tumor group label). Predictions on a patient-level were obtained by selecting the most frequent prediction label of the RFC applied to the PBMCs of the respective patient.
- **g.** Barplot showing the importance of the chrometric features used by RFC to distinguish between the different tumor groups given the chrometric profiles of the PBMCs. The color-coding is done in alignment with Fig. 2 and the top 15 features are shown.

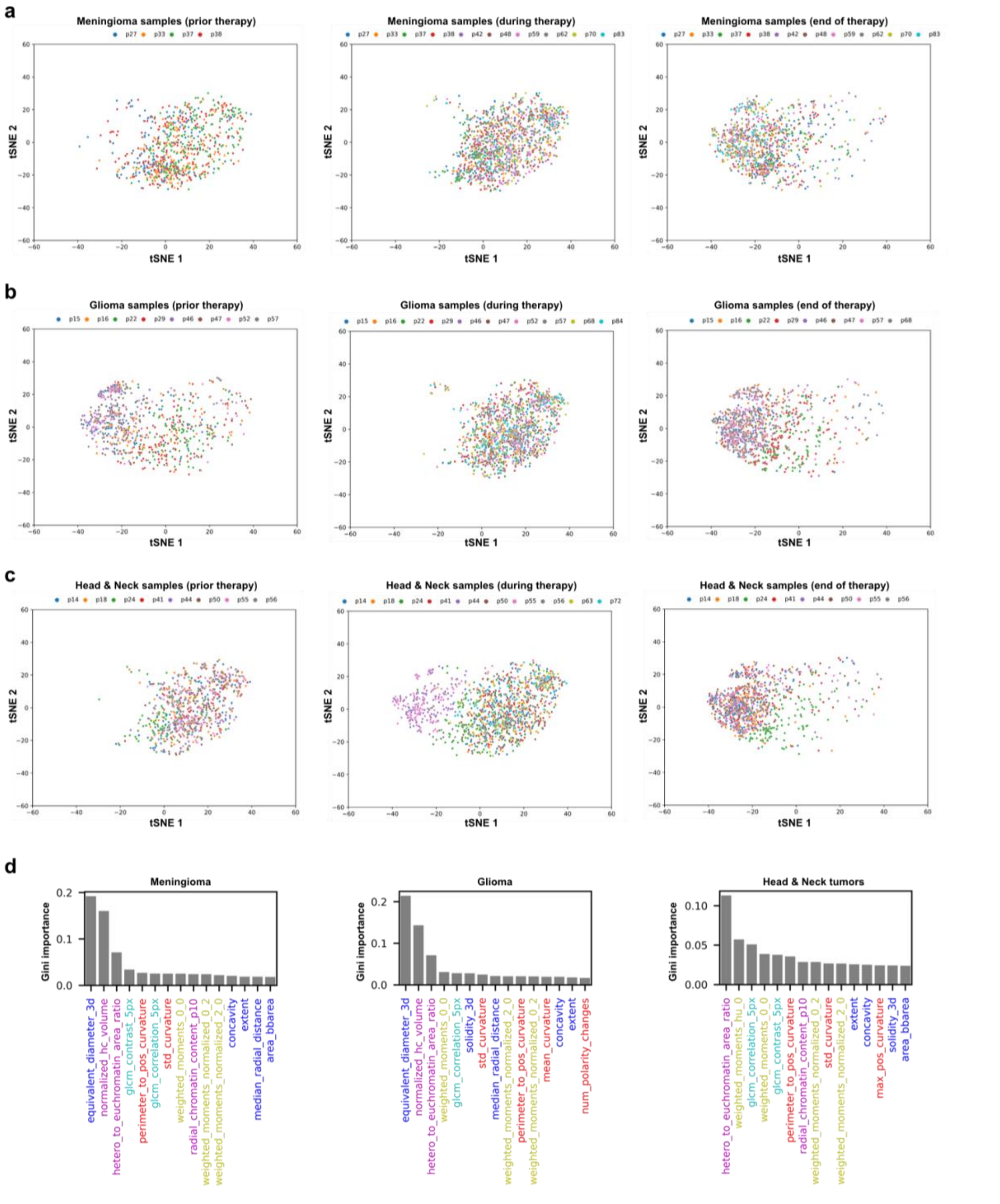

Supplementary Figure 4: Extended analyses of the chromatin organization of PBMCs over the course of proton therapy treatment.

- **a.** Visualization of the chrometric profiles of the PBMCs (n=3,132 in total) of the Meningioma population prior to, during and at the end of proton therapy (left to right) using a tSNE plot. Each point represents a single PBMC which is colored according to the patient sample it originates from.
- **b.** Visualization of the chrometric profiles of the PBMCs (n=3,132 in total) of the Glioma population prior to, during and at the end of proton therapy (left to right) using a tSNE plot. Each point represents a single PBMC which is colored according to the patient sample it originates from.
- **c.** Visualization of the chrometric profiles of the PBMCs (n=3,132 in total) of the Head & Neck tumor population prior to, during and at the end of proton therapy (left to right) using a tSNE plot. Each point represents a single PBMC which is colored according to the patient sample it originates from.
- **d.** Barplot showing the importance of the chrometric features used by RFC to distinguish between the different treatment time points for the Meningioma (right), Glioma (center) and Head and neck tumor populations given the chrometric profiles of the PBMCs. The color-coding is in accordance with Fig. 2 and the top 15 features are shown.

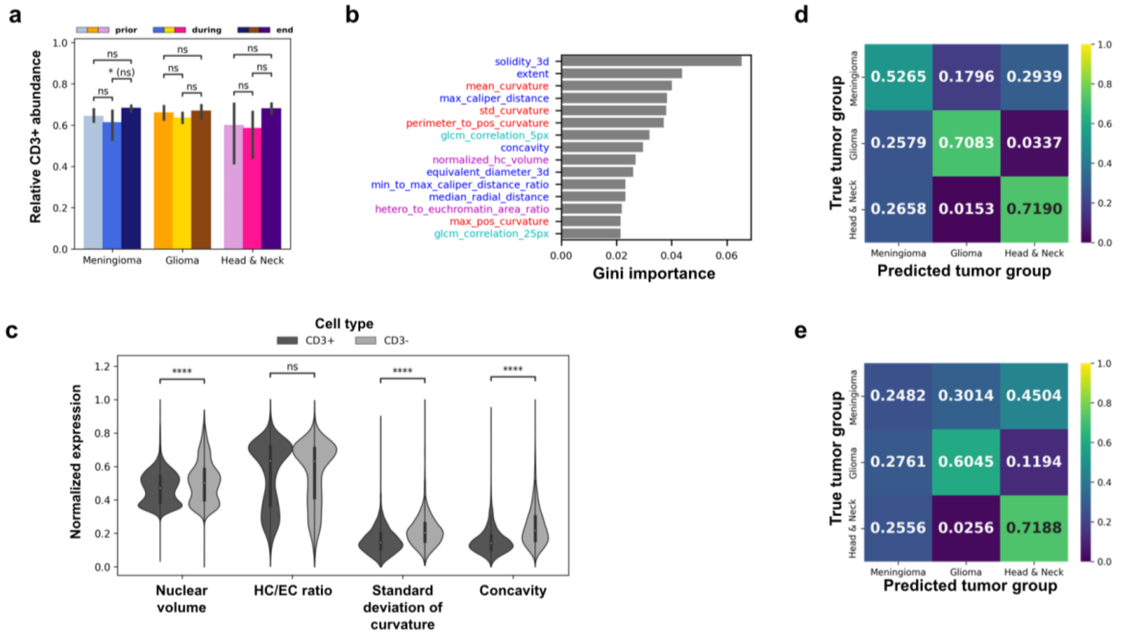

Supplementary Figure 5: Extended analyses of the cell type-specific effects of proton therapy on PBMCs.

- **a.** Bar plot showing the average, relative abundance of CD3+ T-cells across the different patient-specific PBMC populations across the different tumor groups and treatment time points. Error bars reflect one standard deviation. P-values: to be added, Welch's t-test.
- **b.** Barplot showing the importance of the chrometric features used by RFC to distinguish between CD3+ and CD3- PBMCs based on their chrometric profiles. The color-coding is in accordance with Fig. 2 and the top 15 features are shown.
- **c.** Violin plot showing the range-normalized expression of the three most differentially expressed chrometric features between the CD3+ (dark gray) and the CD3- (light gray) PBMCs. P-values are coded as defined in Fig. 3E, two-sided Welch's t-tests.
- **d.** Average of the row-normalized confusion matrices for a RFC evaluated in a leave-one-patient-out cross-validation scheme on the task of classifying the respective tumor group label of PBMC given their chrometric profiles using only CD3+ PBMCs (i.e. T cells) (see Methods). The classifier achieves an average accuracy of 0.67 (+/- 0.30).
- **e.** Average of the row-normalized confusion matrices for a RFC evaluated in a leave-one-patient-out cross-validation scheme on the task of classifying the respective tumor group label of PBMC given their chrometric profiles using only CD3- PBMCs (i.e. non-T cells) (see Methods). Two head and neck patients with less than two CD3+ T cells were excluded prior to the analyses. The classifier achieves an average accuracy of 0.61 (+/- 0.32).

Supplementary Table 1: Overview of the study population forming the control and pan-tumor population.

Ages are given in years. Sex is abbreviated as (M) for male and (F) for female. The preceding chemotherapy column indicates if the patient has undergone chemotherapy prior to the study enrollment.

Patient ID	Age	Sex	Diagnosis	Condition	Preceding chemotherapy
HV01	24	M	healthy	control	n/a
HV02	33	M	healthy	control	n/a
HV03	42	F	healthy	control	n/a
HV04	60	M	healthy	control	n/a
HV05	26	M	healthy	control	n/a
HV06	60	M	healthy	control	n/a
HV07	32	M	healthy	control	n/a
HV08	28	F	healthy	control	n/a
HV09	45	F	healthy	control	n/a
HV10	35	M	healthy	control	n/a
P11	5	F	Embryonal Rhabdomyosarcoma	tumor	yes
P12	53	F	Neuroblastoma	tumor	no
P13	10	M	Hodgkin’s lymphoma	tumor	yes
P14	17	M	Squamous cell carcinoma	tumor	yes
P15	37	F	Astrocytoma	tumor	no
P16	17	M	Glioblastoma	tumor	no
P17	21	F	Hodgkin’s lymphoma	tumor	yes
P18	41	M	Adenoid cystic carcinoma	tumor	no
P19	7	F	Medulloblastoma	tumor	no
P20	29	M	Hemangiopericytoma	tumor	no

Supplementary Table 2: Overview of the study population corresponding to the Meningioma patients.

Ages are given in years. Sex is abbreviated as (M) for male and (F) for female. Chemotherapy indicates if the patient has undergone chemotherapy prior to the study enrollment. The chemotherapy column indicates if patients underwent proton therapy prior, during and/or after proton therapy.

Patient ID	Age	Sex	Diagnosis	Chemotherapy
P27	61	F	Meningioma	none
P33	57	M	Meningioma	none
P37	28	M	Meningioma	none
P38	50	M	Meningioma	none
P42	67	F	Meningioma	none
P48	46	F	Meningioma	none
P59	43	F	Meningioma	none
P62	58	F	Meningioma	none
P70	51	F	Meningioma	none
P83	16	M	Meningioma	none

Supplementary Table 3: Overview of the study population corresponding to the Glioma patients.

Ages are given in years. Sex is abbreviated as (M) for male and (F) for female. Chemotherapy indicates if the patient has undergone chemotherapy prior to the study enrollment. The chemotherapy column indicates if patients underwent proton therapy prior, during and/or after proton therapy.

Patient ID	Age	Sex	Diagnosis	Chemotherapy
P15	37	F	Astrocytoma	none
P16	17	M	Glioblastoma	during, after
P22	38	M	Astrocytoma	before
P29	46	M	Pilocytic Astrocytoma	none
P46	42	F	Oligodendroglioma	none
P47	38	F	Astrocytome	none
P52	19	F	Glioblastoma	none
P57	41	M	Astrocytoma	before, during, after
P68	25	M	Oligodendroglioma	after
P84	27	F	Oligodendroglioma	none

Supplementary Table 4: Overview of the study population corresponding to the head and neck tumor patients.

Ages are given in years. Sex is abbreviated as (M) for male and (F) for female. Chemotherapy indicates if the patient has undergone chemotherapy prior to the study enrollment. The chemotherapy column indicates if patients underwent proton therapy prior, during and/or after proton therapy.

Patient ID	Age	Sex	Diagnosis	Chemotherapy
P14	17	M	Squamous cell carcinoma	before, during
P18	41	M	Adenoid cystic carcinoma	none
P24	41	F	Sinonasal undifferentiated carcinoma	before, during
P41	50	M	Acinar cell carcinoma parotid gland	none
P44	59	M	Squamous cell carcinoma	before, during
P50	79	F	Squamous cell carcinoma	None
P55	53	M	Squamous cell carcinoma	before, during
P56	49	M	Acinar cell carcinoma parotid gland	none
P63	81	M	Acinar cell carcinoma parotid gland	none
P72	63	F	Adenoid cystic carcinoma parotid gland	during
